# Epilepsy surgery of dysembryoplastic neuroepithelial tumors using advanced multitechnologies with combined neuroimaging and electrophysiological examinations^☆^

**DOI:** 10.1016/j.ebcr.2013.06.002

**Published:** 2013-07-27

**Authors:** Jun Shinoda, Kazutoshi Yokoyama, Kazuhiro Miwa, Takeshi Ito, Yoshitaka Asano, Shingo Yonezawa, Hirohito Yano

**Affiliations:** a Chubu Medical Center for Prolonged Traumatic Brain Dysfunction and Section of Neurosurgery, Kizawa Memorial Hospital, Department of Clinical Brain Sciences, Gifu University Graduate School of Medicine, Japan; b Department of Neurosurgery, Gifu University Graduate School of Medicine, Japan

**Keywords:** Dysembryoplastic neuroepithelial tumor, Epilepsy, Positron emission tomography, Electrocorticography, Magnetoencephalography, Tractography

## Abstract

**Purpose:**

We report three cases of dysembryoplastic neuroepithelial tumor (DNT) with intractable epilepsy which were successfully treated with surgery.

**Methods:**

In all cases, technology beyond the routine workup was critical to success. Preoperative magnetic resonance imaging, ^18^F-fluorodeoxyglucose positron emission tomography (PET), ^11^C-methionine-PET, interictal electroencephalography, and intraoperative electrocorticography were utilized in all patients. In individual cases, however, additional procedures such as preoperative magnetoencephalography (Case 1), diffusion tensor fiber tractography, a neuronavigation system, and intraoperative somatosensory-evoked potential (Case 2), and fiber tractography and the neuronavigation-guided fence-post tube technique (Case 3) were instrumental.

**Results:**

In all the cases, the objectives of total tumor resection, resection of the epileptogenic zone, and complete postoperative seizure control and the avoidance of surgical complications were achieved.

**Conclusions:**

Dysembryoplastic neuroepithelial tumor is commonly associated with medically intractable epilepsy, and surgery is frequently utilized. As DNT may arise in any supratentorial and intracortical locations within or near the critical area of the brain, meticulous surgical strategies are necessary to avoid neurological deficits. We demonstrate in the following three cases how adjunct procedures using advanced multitechnologies with neuroimaging and electrophysiological examinations may be utilized to ensure success in DNT surgery.

## 1 Introduction

Dysembryoplastic neuroepithelial tumor (DNT), first described by Daumas-Duport et al. [1], is, under the current World Health Organization (WHO) classification, a low-grade glioneuronal tumor causing intractable complex partial seizures [2]. Complete resolution of seizures in a large percentage of both adult and pediatric patients is achieved with surgery [3], [4], [5], [6], [7], [8]. Though the primary objective of surgery is complete seizure control without anticonvulsant therapy, the prevention of recurrent disease and the diagnosis of malignant transformation are also goals of surgical resection [9]. The widespread surgical treatment of epilepsy due to DNT has, however, been criticized because surgery carries a nonnegligible risk of surgical sequelae including neurological, cognitive, and neuropsychological impairment. Guidelines regarding the preoperative evaluation and intraoperative determination of the extent of resection have not been standardized. Magnetic resonance imaging (MRI), ^11^C-methionine positron emission tomography (MET-PET), and ^18^F-fluorodeoxyglucose (FDG)-PET are routinely used at our institution preoperatively to assess the morphology and metabolism of brain tumors with epileptogenicity in addition to interictal electroencephalography (EEG). Additionally, electrocorticography (ECoG) is also used intraoperatively to detect the epileptogenic zone (EZ).

We report three cases of DNT with intractable epilepsy, successfully treated with surgery, in which not only imaging functional studies but also advanced neurosurgical technologies were critical for planning and supported the role for surgery. These interventions included preoperative magnetoencephalography (MEG) (Case 1), fiber tractography obtained from diffusion tensor imaging (DTI), a neuronavigation system, and intraoperative somatosensory evoked potential (SEP) (Case 2) and fiber tractography and the neuronavigation-guided fence-post tube technique (Case 3) (Table 1).

**Table 1:** Clinical summary of the three cases of DNT.

Case no.	Age (years)	Sex	Preoperative duration of epilepsy	Tumor location	Tumor size (cm)	ME used preope.	ME used intraoperatively	Surgery	Extent of tumor resection	Surgical complications	Engel class
1	43	F	20 years	Left temporal lobe	1.8 × 1.8 × 1.8	MRI EEG MET-PET FDG-PET MEG	ECoG	First	Total	None	I
2	5	F	4 months	Left frontal lobe	3.0 × 3.0 × 4.0	MRI EEG MET-PET FDG-PET TG	ECoG SEP NNS	First	Total	None	I
3	10	F	4 years	Left temporal lobe	6.5 × 5.0 × 4.0	MRI EEG MET-PET FDG-PET TG	ECoG NNGFPT	Second	Total	None	I

ME = medical equipment; MRI = magnetic resonance imaging; EEG = electroencephalography; MET-PET = ^11^C-methionine positron emission tomography; FDG-PET = ^18^F-fluorodeoxyglucose positron emission tomography; MEG = magnetoencephalography; TG = tractography; ECoG = electrocorticography; SEP = somatosensory-evoked potential; NNS = neuronavigation system; NNGFPT = neuronavigation-guided fence-post tube technique.

The medical equipment used in these cases were MRI (Signa, GE Healthcare, Milwaukee, Wisconsin, USA and Achieva 3.0 T, Philips, The Netherlands), PET (GE, Yokokawa Medical System, Hino, Tokyo, Japan and Eminence Stargate, Shimadzu, Kyoto, Japan), neuronavigation system (VectorVision, BrainLab, Munchen, Germany), SEP/EEG/ECoG (Neuropack X1, Nihonkohden, Tokyo, Japan), and MEG (Elekta Neuromag, Helsinki, Finland). Magnetoencephalography in Case 1 was performed at Komaki City Hospital.

## 2 Case report

### 2.1 Case 1

**Fig. 1:**
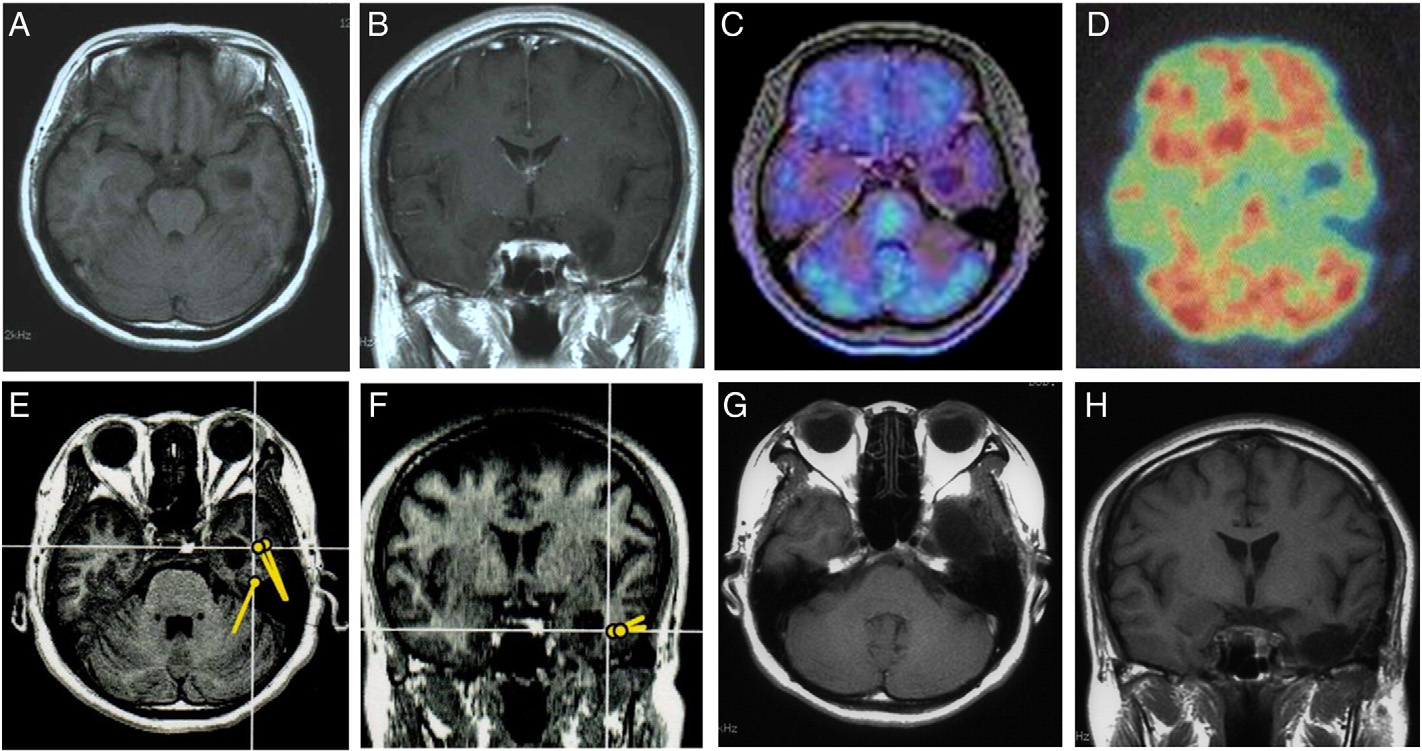
Case 1. T1WI showing a relatively well-demarcated, small hypointensity mass lesion in the left medial temporal lobe in axial (A) and coronal (B) images. The tumor shows low uptake in MET-PET (fused with T1WI) (C) and hypo-uptake in FDG-PET (D). Preoperative interictal MEG shows clustered dipoles in the region lateral to the tumor (E and F). Postoperative T1WI showed the tumor to be totally resected (G and H).

**Fig. 2:**
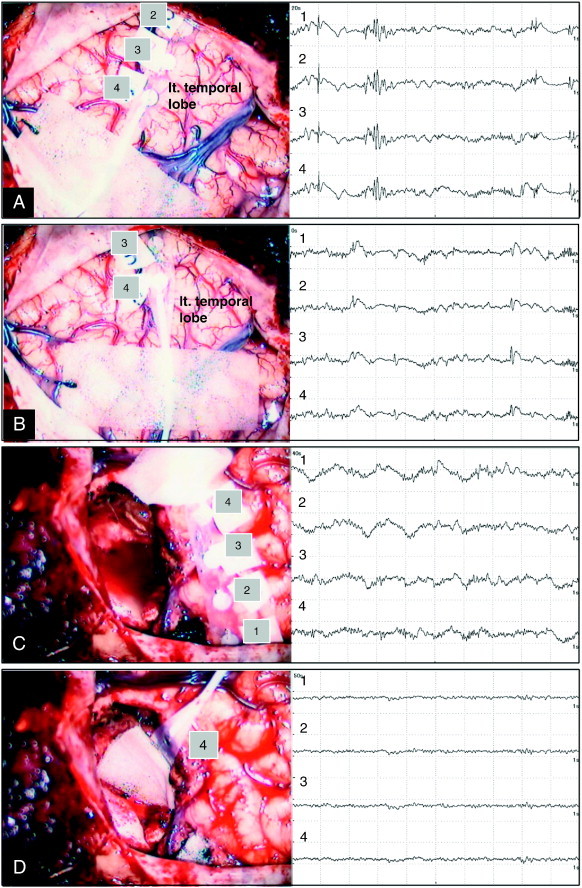
Case 1. Intraoperative ECoG prior to lesion resection showing interictal spikes, with representative ECoGs shown from different two sites (A and B). Epileptiform discharges which emerged on ECoG before lesion resection disappeared after lesion resection as shown on representative ECoGs from two different sites (C and D).

The patient, a 43-year-old woman, presented with a 20-year history of complex partial seizures occasionally followed by generalized tonic–clonic convulsive seizures. Despite treatment for over two years with multiple anticonvulsants, she continued to have seizures several times a month. Her MRI showed a relatively well-demarcated, small, mass lesion (1.8 × 1.8 × 1.8 cm) in the left medial temporal lobe, which presented with hypointensity on a T1-weighted image (T1WI), hyperintensity on T2WI, hypointensity with a surrounding high intensity ring in fluid attenuated inversion recovery (FLAIR), no gadolinium (Gd) enhancement, low uptake in MET-PET, and hypo-uptake in FDG-PET (Figs. 1A, B, C, and D). Preoperative interictal EEG did not show any significant epileptogenic activity. Preoperative interictal MEG, however, showed clustered dipoles in the region lateral to the tumor (Figs. 1E and F). A combined total tumor resection and left anterior temporal lobectomy was performed. Intraoperative ECoG monitoring was used to verify complete resection of the EZ, which was defined as the peritumoral region with interictal spikes on the intraoperative ECoG (Figs. 1G and H, 2A and B). Epileptiform discharges, which emerged on ECoG before resection of the lesion, disappeared after lesion resection (Figs. 2C and D). There were no surgical complications. The tumor was histologically diagnosed as a DNT. The patient was continued on anticonvulsants for 12 months postoperatively. Following cessation of the anticonvulsants, she remained seizure-free as of nine years following surgery. Postoperative interictal EEGs have not shown any significant epileptogenic activity (Engel class I [9]).

### 2.2 Case 2

**Fig. 3:**
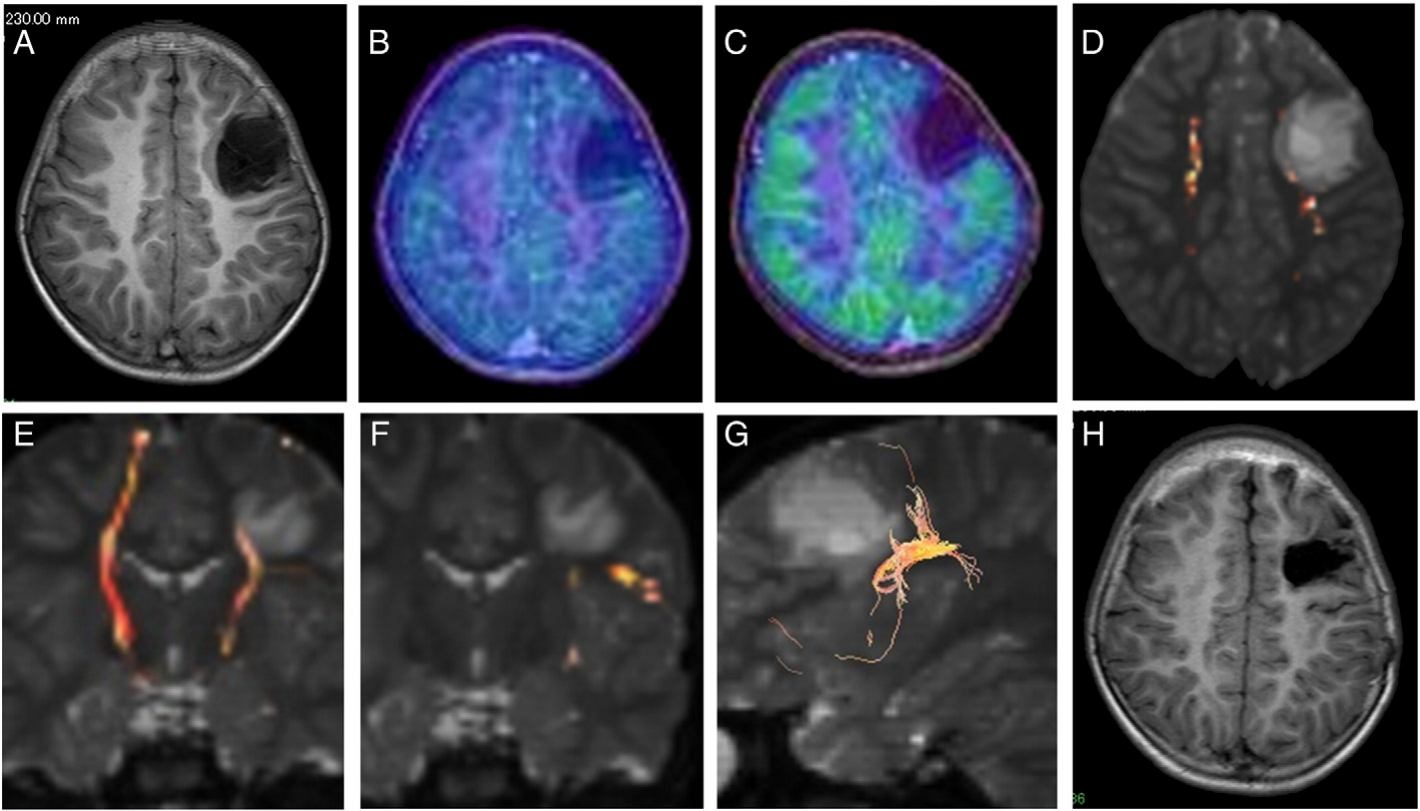
Case 2. T1WI showing a well-demarcated hypointensity mass lesion in the left frontal lobe (A). The tumor shows low uptake in MET-PET (fused with T1WI) (B) and hypo-uptake in FDG-PET (fused with T1WI) (C). Fiber tractography showing that the left pyramidal tract is located posteromediocaudal to the tumor (D and E) and that the left arcuate fasciculus is located caudolateral to the tumor (F and G). Postoperative T1WI showed the tumor to be totally resected (H).

**Fig. 4:**
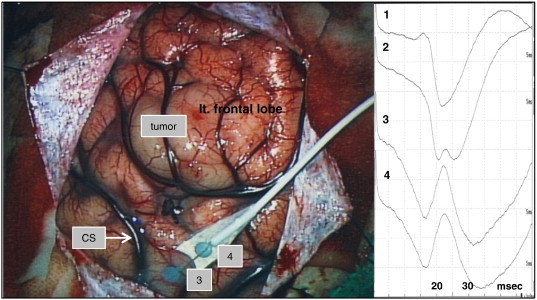
Case 2. The left central sulcus was identified using intraoperative SEP. CS = central sulcus.

**Fig. 5:**
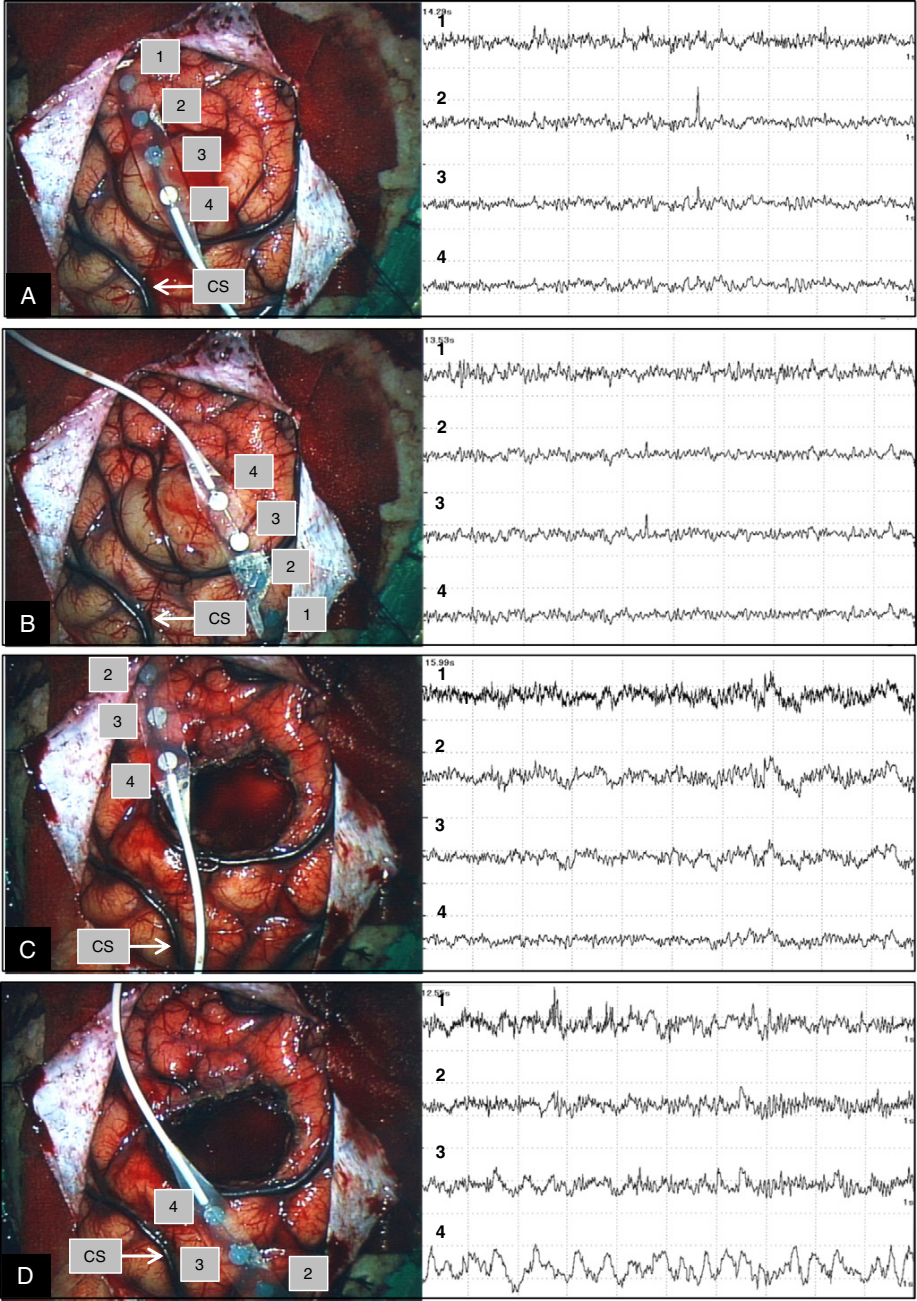
Case 2. Intraoperative ECoG before the lesion resection showing interictal spikes, and the representative ECoGs from two different sites (A and B). Epileptiform discharges which emerged on ECoG before lesion resection disappeared after lesion resection, and the representative ECoGs from two different sites are shown (C and D). CS = central sulcus.

The patient, a five-year-old girl, presented a four-month history of complex partial seizures occurring a few times a day. Anticonvulsants reduced the seizure frequency to a few times a week. Her MRI showed a relatively well-demarcated mass lesion (3.0 × 3.0 × 4.0 cm) in the left frontal lobe extending to the left lateral ventricle wall, which presented with hypointensity on T1WI, hyperintensity on T2WI, hypointensity with a surrounding high intensity irregular ring on FLAIR, no Gd enhancement, low uptake in MET-PET, and hypo-uptake in FDG-PET (Figs. 3A, B, and C). Preoperative interictal EEG showed frequent epileptiform spike discharges on the left frontal region. Fiber tractography showed that the left pyramidal tract lay just posteromediocaudal to the tumor (Figs. 3D and E) and that the left arcuate fasciculus lay just caudolateral to the tumor (Figs. 3F and G). In the surgery, at first, the left central sulcus was identified using intraoperative SEP (Fig. 4). A total tumor resection with careful resection of the EZ, which was defined as the peritumoral regions with interictal spikes on the intraoperative ECoG, was performed using a neuronavigation system under monitoring of the intraoperative ECoG (Figs. 3H, 5A and B). Epileptiform discharges, which emerged on ECoG before the lesion was resected, completely disappeared after lesion resection (Figs. 5C and D). She had no surgical complications. The tumor was histologically diagnosed as a DNT. She was continued on anticonvulsants for 12 months postoperatively. She remains seizure-free and off anticonvulsants as of her most recent follow-up three years after surgery. Her postoperative interictal EEG has not shown any significant epileptogenic activity (Engel class I [9]).

### 2.3 Case 3

**Fig. 6:**
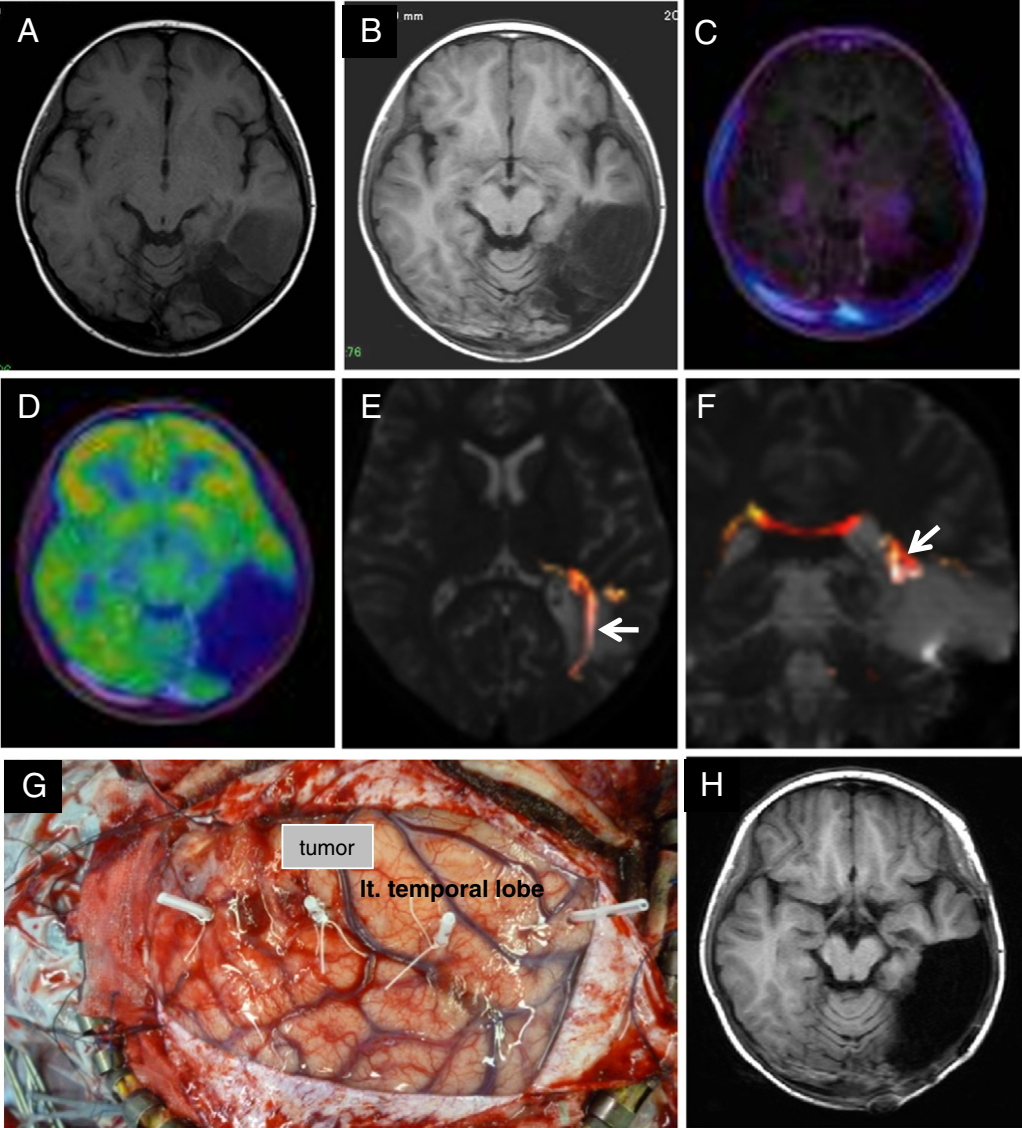
Case 3. T1WI after the first surgery showing a partially resected, well-demarcated, hypointensity mass lesion in the left posterior temporal lobe (A). Two years later, T1WI showing tumor progression (B). The tumor showed low uptake in MET-PET (fused with T1WI) (C) and hypo-uptake in FDG-PET (fused with T1WI) (D). Fiber tractography showed the left visual tract (arrow) to be mediorostral to the tumor (E and F). This is a photograph showing the neuronavigation-guided fence-post tube technique used in the surgery (G). Postoperative T1WI showed that the tumor was totally resected (H).

**Fig. 7:**
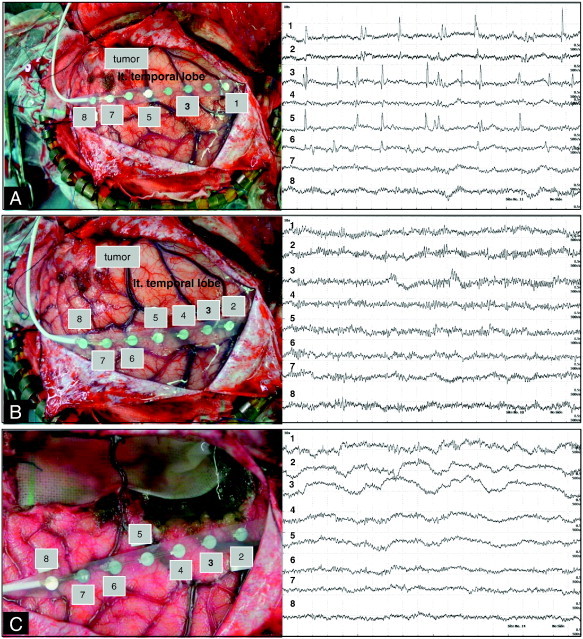
Case 3. Intraoperative ECoG before lesion resection showing interictal spikes and the representative ECoGs from two different sites (A and B). Epileptiform discharges which emerged on ECoG before lesion resection disappeared after lesion resection on the representative ECoG (C).

The patient, a 10-year-old girl, presented with a history of surgery for DNT in the left posterior temporal lobe. She had developed complex partial seizures refractory to anticonvulsants and had seizures a few times a month before the surgery. She underwent a partial resection of the tumor in a community hospital at the age of eight years, and the tumor was diagnosed as a DNT histologically (Fig. 6A). After surgery, she was seizure-free for 12 months on anticonvulsant medication; however, seizures recurred a few times per day. She was referred to our hospital, and her MRI showed a relatively well-demarcated mass lesion (6.5 × 5.0 × 4.0 cm) in the left posterior temporal lobe with a cavity corresponding to the prior area of resection in the posterior portion of the tumor. The tumor presented with hypointensity on T1WI, hyperintensity on T2WI, hypointensity with a surrounding irregular high intensity area in the white matter on FLAIR, and no Gd enhancement (Fig. 6B). The MRI revealed tumor progression, and the tumor intensities were the same as on the prior preoperative imaging (Figs. 6A and B). The tumor showed low uptake in MET-PET and hypo-uptake in FDG-PET (Figs. 6C and D). Preoperative interictal EEG showed frequent epileptiform spike discharges on the left occipitotemporal region. The previous surgery had resulted in right upper quadrant hemianopsia. Fiber tractography showed that the residual left visual tract lay just mediorostral to the tumor (Figs. 6E and F). We performed a complete total tumor resection with careful resection of the EZ, which was defined as the peritumoral region with interictal spikes on the intraoperative ECoG, using a neuronavigation-guided fence-post tube technique with monitoring of intraoperative ECoG (Figs. 6G and H, 7A and B). Epileptiform discharges which emerged on the ECoG completely disappeared after lesion resection (Fig. 7C). She had no surgical complications and exhibited no worsening of the visual field deficit. The tumor was diagnosed as a DNT histologically without malignant transformation. She was maintained on anticonvulsants and had no recurrence five months postoperatively. Postoperative interictal EEG did not show any significant epileptogenic activity (Engel class I [9]).

## 3 Discussion

The goals of surgery for DNT include not only complete tumor resection but also complete seizure control by resecting the EZ, while avoiding surgical sequela [10]. Imaging and functional studies are indispensable adjuncts to meticulous surgical techniques for achieving these goals. In addition to preoperative interictal EEG and MRI, preoperative FDG-PET, MET-PET, and intraoperative ECoG have been used routinely for surgical planning for resection of brain lesions with epileptogenicity such as DNT in our hospital.

Characteristics of DNT, such as a well-demarcated margin, benign clinical course, indolent biology, and the frequent association of a peritumoral EZ, are conducive to surgical management. The preoperative diagnosis of a DNT facilitates the assemblage of intraoperative monitoring equipment necessary for precisely excising the EZ. Epileptogenic brain tumors which should be differentiated from DNT include diffuse astrocytoma, oligodendroglioma, pleomorphic xanthoastrocytoma, pilocytic astrocytoma, gangliocytoma, and ganglioglioma. Some may be easily differentiated from DNT using a conventional CT and/or MRI; however, others cannot precisely be differentiated because of their similar radiological features. ^11^C-methionine positron emission tomography has improved the radiological diagnosis of brain tumors [11]. Among these brain tumors, a finding of low uptake in MET-PET is strongly suggestive of a DNT, and the diagnosis becomes more reliable if associated with corresponding morphology on CT and/or MRI [12], [13]. The hypo-uptake in FDG-PET is another characteristic of DNT, which suggests not only the hypometabolism of the tumor but also a lack of functional activity as the normal brain within the lesion. It is this lack of functional activity which permits the complete excision of the tumor without a subsequent postoperative neurological deficit.

Intraoperative ECoG is an essential tool for DNT surgery and is utilized in most surgical procedures for epilepsy [3], [4], [14], [15], [16], [17]. Localization of the EZ associated with a DNT is still controversial, and results are variable. Recently, Chassoux et al. [18] have proposed that three distinct histologic subtypes of DNT are distinguishable based on MRI features. Using MRI, DNTs are classified as type 1 which is cystic/polycystic-like, well-delineated, and strongly hypointense in T1WI; type 2 which is nodular-like and heterogeneous; and type 3 which is dysplastic-like, iso/hypointense in T1WI, with poor delineation and gray–white matter blurring. Resection of type 1 tumors, in which the tumor and the EZ are colocalized, will generally definitively treat the epilepsy. Types 2 and 3, however, require a more extensive resection that includes both the tumor and the perilesional cortex. For example, for type 3 tumors in the temporal lobe, an anterior temporal lobectomy must be considered. Case 2 is a type 1 example and Cases 1 and 3 are type 3 examples. Such a detailed imaging study is informative for preoperative surgical planning for seizure control; however, this is not always helpful to address in delineating the EZ precisely in individual cases. To achieve the objective of seizure control, intraoperative ECoG monitoring is still needed to determine the extent of resection of the lesion including the EZ, although the spike-chasing using ECoG is partly debatable and controversial.

For some DNT, preoperative CT, MRI, interictal EEG, PET studies, and intraoperative ECoG are usually sufficient to achieve seizure control with surgery; however, for others, these are not sufficient. In Case 1, the preoperative interictal EEG did not show any significant epileptogenic activity. Dysembryoplastic neuroepithelial tumor in the temporal lobe, like other epileptic lesions in deep brain structures, often presents with no significant epileptogenic activity in preoperative EEG. In these cases, preoperative chronic subdural electrode recording (CSDER) is strongly recommended to confirm whether the tumor truly has epileptogenicity and to evaluate the extent of the epileptogenic lesion [3], [19]. Magnetoencephalography is a sophisticated medical device which can detect subtle brain activity and the epileptogenicity of lesions. Its clinical role for detecting epileptogenicity preoperatively may replace that of CSDER in those cases with visible lesions on neuroimaging because MEG, unlike CSDER, is noninvasive [20], [21], [22], [23]. In Case 1, MEG was used instead of CSDER.

The DNT in Case 2 was adjacent to the motor cortex and the language pathway. Epileptogenicity was detectable in the tumor by preoperative EEG. Complex partial seizures observed in this case were likely attributable to tumor extension to the left cingulate gyrus. In this case, treatment planning focused on avoiding damage to the motor cortex and the language pathway while aiming for total resection of the tumor with the EZ. In such a case, the combination of fiber tractography, neuronavigation system, and SEP is a valuable adjunct to ECoG and imaging studies [24], [25], [26], [27], [28], [29]. Preoperative fiber tractography that revealed the topographical relationship between the left pyramidal tract and the tumor and also between the left arcuate fasciculus and the tumor was informative for safe tumor resection. The neuronavigation system and intraoperative SEP aided in the identification of the left central sulcus during the surgery. These interventions were indispensable for preventing surgical complications.

Case 3 had a recurrent large DNT adjacent to the left visual tract, in which epileptogenicity was detectable near the tumor by preoperative EEG. The patient had already had a right upper quadrant hemianopsia from the previous surgery. Another clinicopathological concern in this case was the possibility of malignant transformation of the tumor in terms of its rapid regrowth. Dysembryoplastic neuroepithelial tumor is considered benign and tends to not recur even after a partial resection [1], [2]. There are, however, some reported cases of malignant transformation and tumor regrowth [30], [31], [32], [33]. To achieve clinical benefit in this patient, the goal of surgery was complete excision of the tumor including malignant parts and EZ without worsening the neurological deficit. Total tumor resection would prevent further regrowth and enable the complete histopathological evaluation of the tumor in addition to seizure control. Tumor resection was successfully performed using the neuronavigation-guided fence-post tube technique in this case. The neuronavigation-guided fence-post technique is superior to ordinary image-guided neuronavigation for larger tumors because intraoperative structural distortion of the brain due to brain shift is more likely during resection of larger tumors [34], [35]. Preoperative fiber tractogram, revealing the topographical relationship between the left visual tract and the tumor, was informative for placing fence-posts to define the excision margins. No histological evidence of malignant foci was found in the resected lesion including the tumor. Recently, another case of progressive DNT on MRI in a pediatric patient, in which the tumor showed no histological evidence of malignancy, was reported by Preuss et al. [36]. In such cases, tumor growth is attributed to an increase in the mucinous substance in the myxoid matrix. A further careful follow-up is needed for checking tumor and seizure recurrences in Case 3 because of the short period of follow-up after the second surgery.

## 4 Conclusions

Although DNT frequently results in epilepsy that is unresponsive to medical therapy, DNT-associated epilepsy is highly curable with surgery. Dysembryoplastic neuroepithelial tumor may arise in any supratentorial or intracortical location within or near critical areas of the brain. Resection, therefore, must be carefully planned both pre- and intraoperatively and requires meticulous technique. Goals of therapy include not only completely resecting the tumor to avoid recurrence or progression but also excising the entire associated EZ. We have shown that when imaging and functional studies are utilized concurrently with advanced neurosurgical technologies for operative planning, excellent surgical outcomes result.

## Conflict of interest

None of the authors has any conflict of interest to disclosure.

## References

[1] Daumas-Duport C., Scheithauer B.W., Chodkiewicz J.P., Laws E.R., Vedrenne C. (1988). Dysembryoplastic neuroepithelial tumor: a surgically curable tumor of young patients with intractable partial seizures. Report of thirty-nine cases. Neurosurgery.

[2] Daumas-Duport C., Pietsch T., Hawkins C., Shankar S.K., Louis D.N., Ohgaki H., Wiestler O.D., Cavenee W.K. (2007). WHO classification of tumours of the central nervous system.

[3] Sandberg D.I., Ragheb J., Dunoyer C., Bhatia S., Olavarria G., Morrison G. (2005). Surgical outcomes and seizure control rates after resection of dysembryoplastic neuroepithelial tumors. Neurosurg Focus.

[4] Chan C.H., Bittar R.G., Davis G., Kalnins R.M., Fabinyi G.C.A. (2006). Long-term seizure outcome following surgery for dysembryoplastic neuroepithelial tumor. J Neurosurg.

[5] Minkin K., Klein O., Mancini J., Lena G. (2008). Surgical strategies and seizure control in pediatric patients with dysembryoplastic neuroepithelial tumors: a single-institution experience. J Neurosurg Pediatr.

[6] Bilginer B., yalnizoglu D., Soylemezoglu F., Turanli G., Cila A., Topçu M. (2009). Surgery for epilepsy in children with dysembryoplastic neuroepithelial tumor: clinical spectrum, seizure outcome, neuroradiology, and pathology. Childs Nerv Syst.

[7] Spalice A., Ruggieri M., Grosso S., Verrotti A., Polizzi A., Margo G. (2010). Dysembryoplastic neuroepithelial tumors: a prospective clinicopathologic and outcome study of 13 children. Pediatr Neurol.

[8] Chang E.F., Christie C., Sullivan J.E., Garcia P.A., Tihan T., Gupta N. (2010). Seizure control outcomes after resection of dysembryoplastic neuroepithelial tumor in 50 patients. J Neurosurg Pediatr.

[9] Engel J., Van Ness P.C., Rasmusen T.B., Ojemann L.M., Engel J. (1993). Surgical treatment of the epilepsies.

[10] O'Brien D.F., Farrell M., Delanty N., Traunecker H., Perrin R., Smyth M.D. (2007). The Children's Cancer and Leukaemia Group guidelines for the diagnosis and management of dysembryoplastic neuroepithelial tumors. Br J Neurosurg.

[11] Kato T., Shinoda J., Nakayama N., Miwa K., Okumura A., Yano H. (2008). Metabolic assessment of gliomas using carbon-11 methionine, fluorine-18 fluorodeoxyglucose, and carbon-11 choline positron-emission tomography. AJNR Am J Neuroradiol.

[12] Maehara T., Nariai T., Arai N., Kawai K., Shimizu H., Ishii K. (2004). Usefulness of [^11^C] methionine PET in the diagnosis of dysembryoplastic neuroepithelial tumor with temporal lobe epilepsy. Epilepsia.

[13] Rosenberg D.S., Demarquay G., Jouvet A., Le Bars D., Streichenberger N., Sindou M. (2005). [^11^C]-methionine PET: dysembryoplastic neuroepithelial tumours compared with other epileptogenic brain neoplasms. J Neurol Neurosurg Psychiatry.

[14] Kameyama S., Fukuda M., Tomikawa M., Morota N., Oishi M., Wachi M. (2001). Surgical strategy and outcomes for epileptic patients with focal cortical dysplasia or dysembryoplastic neuroepithelial tumor. Epilepsia.

[15] Nolan M.A., Sakuta R., Chuang N., Otsubo H., Rutka J.T., Snead O.C. (2004). Dysembryoplastic neuroepithelial tumors in childhood. Long-term outcome and prognostic features. Neurology.

[16] Takahashi A., Hong S.C., Seo D.W., Hong S.B., Lee M., Suh Y.L. (2005). Frequent association of cortical dysplasia in dysembryoplastic neuroepithelial tumor treated by epilepsy surgery. Surg Neurol.

[17] Lee J., Lee B.L., Joo E.Y., Seo D.W., Hong S.B., Hong S.C. (2009). Dysembryoplastic neuroepithelial tumors in pediatric patients. Brain Dev.

[18] Chassoux F., Rodrigo S., Mellerio C., Landré E., Miquel C., Turak B. (2012). Dysembryoplastic neuroepithelial tumors: an MRI-based scheme for epilepsy surgery. Neurology.

[19] Seo D.W., Hong S.B. (2003). Epileptogenic foci on subdural recording in intractable epilepsy patients with temporal dysembryoplastic neuroepithelial tumor. J Korean Med Sci.

[20] Medvedovsky M., Taulu S., Gaily E., Metsähonkala E.L., Mäkelä JPm Ekstein D., Kipervasser S. (2012). Sensitivity and specificity of seizure-onset zone estimation by ictal magnetoencephalography. Epilepsia.

[21] Kim H., Lim B.C., Jeong W., Kim J.S., Chae J.H., Kim K.J. (2012). Magnetoencephalography in pediatric lesional epilepsy surgery. J Korean Med.

[22] Fujiwara H., Greiner H.M., Hemasilpin N., Lee K.H., Holland-Bouley K., Arthur T. (2012). Ictal MEG onset source localization compared to intracranial EEG and outcome: improved epilepsy presurgical evaluation in pediatrics. Epilepsy Res.

[23] Kakisaka Y., Wang Z.I., Mosher J.C., Nair D.R., Alexopoulos A.V., Burgess R.C. (2012). Magnetoencephalography's higher sensitivity to epileptic spikes may elucidate the profile of electroencephalographically negative epileptic seizures. Epilepsy Behav.

[24] Castellano A., Bello L., Michelozzi C., Gallucci M., Fava E., Iadanza A. (2012). Role of diffusion tensor magnetic resonance tractography in predicting the extent of resection in glioma surgery. Neuro Oncol.

[25] Fernandez-Miranda J.C., Pathak S., Engh J., Jarbo K., Verstynen T., Yeh F.C. (2012). High-definition fiber tractography of the human brain: neuroanatomical validation and neurosurgical applications. Neurosurgery.

[26] Jung T.Y., Jung S., Kim I.Y., Park S.J., Kang S.S., Kim S.H. (2006). Application of neuronavigation system to brain tumor surgery with clinical experience of 420 cases. Minim Invasive Neurosurg.

[27] Tanaka Y., Nariai T., Momose T., Aoyagi M., Maehara T., Tomori T. (2009). Glioma surgery using a multimodal navigation system with integrated metabolic images. J Neurosurg.

[28] Suzuki A., Yasui N. (1992). Intraoperative localization of the central sulcus by cortical somatosensory evoked potentials in brain tumor. Case report. J Neurosurg.

[29] Hayashi Y., Nakada M., Kinoshita M., Hamada J.I. (2013). Functional reorganization in the patient with progressing glioma of pure primary motor cortex: a case report with special reference to the topographic central sulcus definition by SEP. World Neurosurg.

[30] Hammond R.R., Duggal N., Woulfe J.M., Girvin J.P. (2000). Malignant transformation of a dysembryoplastic neuroepithelial tumor: case report. J Neurosurg.

[31] Rushing E.J., Thompson L.D., Mena H. (2003). Malignant transformation of a dysembryoplastic neuroepithelial tumor after radiation and chemotherapy. Ann Diagn Pathol.

[32] Sampetrean O., Maehara T., Arai N., Nemoto T. (2006). Rapidly growing dysembryoplastic neuroepithelial tumor: case report. Neurosurgery.

[33] Thom M., Toma A., An S., Martinian L., Hadjivassiliou G., Ratilal B. (2011). One hundred and one dysembryoplastic neuroepithelial tumors: an adult epilepsy series with immunohistochemical, molecular genetic, and clinical correlations and a review of the literature. J Neuropathol Exp Neurol.

[34] Yoshikawa K., Kajiwara K., Morioka J., Fujii M., Tanaka N., Fujisawa H. (2006). Improvement of functional outcome after radical surgery in glioblastoma patients: the efficacy of a navigation-guided fence-post procedure and neurophysiological monitoring. J Neurooncol.

[35] Kajiwara K., Yoshikawa K., Ideguchi M., Nomura S., Fujisawa H., Akimura T. (2010). Navigation-guided fence-post tube technique for resection of a brain tumor: technical note. Minim Invasive Neurosurg.

[36] Preuss M., Nestler U., Zühlke C.J., Kuchelmeister K., Neubauer B.A., Jödicke A. (2010). Progressive biological behavior of a dysembryoplastic neuroepithelial tumor. Pediatr Neurosurg.

